# miR-199a and miR-497 Are Associated with Better Overall Survival due to Increased Chemosensitivity in Diffuse Large B-Cell Lymphoma Patients

**DOI:** 10.3390/ijms160818077

**Published:** 2015-08-05

**Authors:** Katharina Troppan, Kerstin Wenzl, Martin Pichler, Beata Pursche, Daniela Schwarzenbacher, Julia Feichtinger, Gerhard G. Thallinger, Christine Beham-Schmid, Peter Neumeister, Alexander Deutsch

**Affiliations:** 1 Division of Hematology, Department of Internal Medicine, Medical University Graz, 8036 Graz, Austria; E-Mails: kerstin.wenzl@medunigraz.at (K.W.); beata.pursche@klinikum-graz.at (B.P.); peter.neumeister@medunigraz.at (P.N.); alexander.deutsch@medunigraz.at (A.D.); 2 Department of Experimental Therapeutics, the University of Texas MD Anderson Cancer Center, Houston, TX 77030, USA; E-Mail: mpichler@mdanderson.org; 3 Division of Oncology, Department of Internal Medicine, Medical University Graz, 8036 Graz, Austria; E-Mail: daniela.schwarzenbacher@medunigraz.at; 4 Bioinformatics, Institute for Knowledge Discovery, Graz University of Technology, 8010 Graz, Austria; E-Mails: julia.feichtinger@tugraz.at (J.F.); gerhard.thallinger@tugraz.at (G.G.T.); 5 Omics Center Graz, BioTechMed Graz, 8010 Graz, Austria; 6 Department of Pathology, Medical University Graz, 8036 Graz, Austria; E-Mail: christine.beham@medunigraz.at

**Keywords:** miRNA-199a, miRNA-497, DLBCL, prognosis, chemosensitivity

## Abstract

Micro-RNAs (miRNAs) are short non-coding single-stranded RNA molecules regulating gene expression at the post-transcriptional level. miRNAs are involved in cell development, differentiation, apoptosis, and proliferation. miRNAs can either function as tumor suppressor genes or oncogenes in various important pathways. The expression of specific miRNAs has been identified to correlate with tumor prognosis. For miRNA expression analysis real-time PCR on 81 samples was performed, including 63 diffuse large B-cell lymphoma (DLBCL, 15 of germinal center B-cell like subtype, 17 non germinal center B-cell, 23 transformed, and eight unclassified) and 18 controls, including nine peripheral B-cells, 5 germinal-center B-cells, four lymphadenitis samples, and 4 lymphoma cell lines (RI-1, SUDHL4, Karpas, U2932). Expression levels of a panel of 11 miRNAs that have been previously involved in other types of cancer (miR-15b_2, miR-16_1*, miR-16_2, miR-16_2*, miR-27a, miR-27a*, miR-98-1, miR-103a, miR-185, miR-199a, and miR-497) were measured and correlated with clinical data. Furthermore, cell lines, lacking miR-199a and miR-497 expression, were electroporated with the two respective miRNAs and treated with standard immunochemotherapy routinely used in patients with DLBCL, followed by functional analyses including cell count and apoptosis assays. Seven miRNAs (miR-16_1*, miR-16_2*, miR-27a, miR-103, miR-185, miR-199, and miR-497) were statistically significantly up-regulated in DLBCL compared to normal germinal cells. However, high expression of miR-497 or miR-199a was associated with better overall survival (*p* = 0.042 and *p* = 0.007). Overexpression of miR-199a and miR-497 led to a statistically significant decrease in viable cells in a dose-dependent fashion after exposure to rituximab and various chemotherapeutics relevant in multi-agent lymphoma therapy. Our data indicate that elevated miR-199a and miR-497 levels are associated with improved survival in aggressive lymphoma patients most likely by modifying drug sensitivity to immunochemotherapy. This functional impairment may serve as a potential novel therapeutic target in future treatment of patients with DLBCL.

## 1. Introduction

Diffuse large B-cell lymphoma (DLBCL) is the most common form of adult lymphoma, accounting for 30% to 40% of newly diagnosed non-Hodgkin lymphoma (NHL) [1]. DLBCL represents a heterogeneous group of tumors with a high variance of genetic abnormalities, clinical features, response to treatment, and prognosis [2]. An immunochemotherapy regimen, consisting of rituximab, cyclophosphamide, doxorubicin, vincristin, and prednisone (R-CHOP) has been established as the standard treatment of patients with DLBCL [3]. With this therapy, DLBCL is considered a curable disease, even in advanced stages. Despite improvements in therapy, in approximately one-third of patients with advanced-stage DLBCL, the disease is refractory to therapy or will relapse [4]. Although new prognostic tools, like the revised International Prognostic Index (R-IPI) [5] or the National Comprehensive Cancer Network (NCCN)-IPI [6] have been introduced for prediction of outcome for these patients, there still remain numerous patients with difficulty in predicting the clinical course. More refined differentiation methods in screening for patients with poor prognosis are necessary to adapt targeted therapy for these patients [7,8].

miRNAs are 19–24 nucleotides long non-coding RNAs that fine tune gene expression through sequence similarities with putative target mRNAs, mostly 3′-untranslated regions of target genes [9]. miRNAs play a role in various biological processes including cancer development [10]. Genes coding for miRNAs are frequently located at fragile sites or genomic regions typically associated with cancer [11]. In 2002, for the first time, Calin* et al.* identified a direct association of deregulation in miR-15 and miR-16 expression, and the development of chronic lymphocytic leukemia [12]. Following the initial findings, meanwhile, many other miRNAs have been identified which participate in cancer development in various tumor types [13]. Additionally, many studies explored the potential clinical application of miRNAs as diagnostic or therapeutic tools for patients with cancer [14,15,16,17,18,19].

A possible tumor suppressive role of miR-199a and miR-497 has been identified in single studies [20,21]. Given the promising potential of these regulatory miRNAs, their role in different cancer types has to be elucidated. Until now, the expression pattern of miR-199a and miR-497 in human DLBCL and its prognostic significance have not been investigated systematically. To clarify their role in DLBCL patients, expression analyses of miR-199a and miR-497 and their influence on patients’ survival were performed. Furthermore, we evaluated the role of the miRNAs in the response of DLBCL cell lines to standard immunochemotherapy.

## 2. Results

### 2.1. Overexpression of miR-199a and miR-497 Are Associated with Better Overall Survival

Clinicopathological parameters of the study population are summarized in Table 1.

**Table 1:** Main clinical features of 58 patients.

Feature	Findings	
Sex M/F	30/28	
Age, median (range), years	65 (28–89)	
Extranodal involvement, %	17	
Stage, Ann Arbor	Stage I 10	Stage III 22
	Stage II 15	Stage IV 11
Bone marrow infiltration, %	25	
R-IPI risk, %	1 (Low) 35	3 (High intermediate) 21
	2 (Low intermediate) 28	4 (High) 16
PFS, median (range), months	56 (0–114)	
OS, median (range), months	44 (0–119)	

M: male; F: female; R-IPI: revised International Prognostic Index; PFS: Progression free survival; OS: Overall survival.

**Figure 1:**
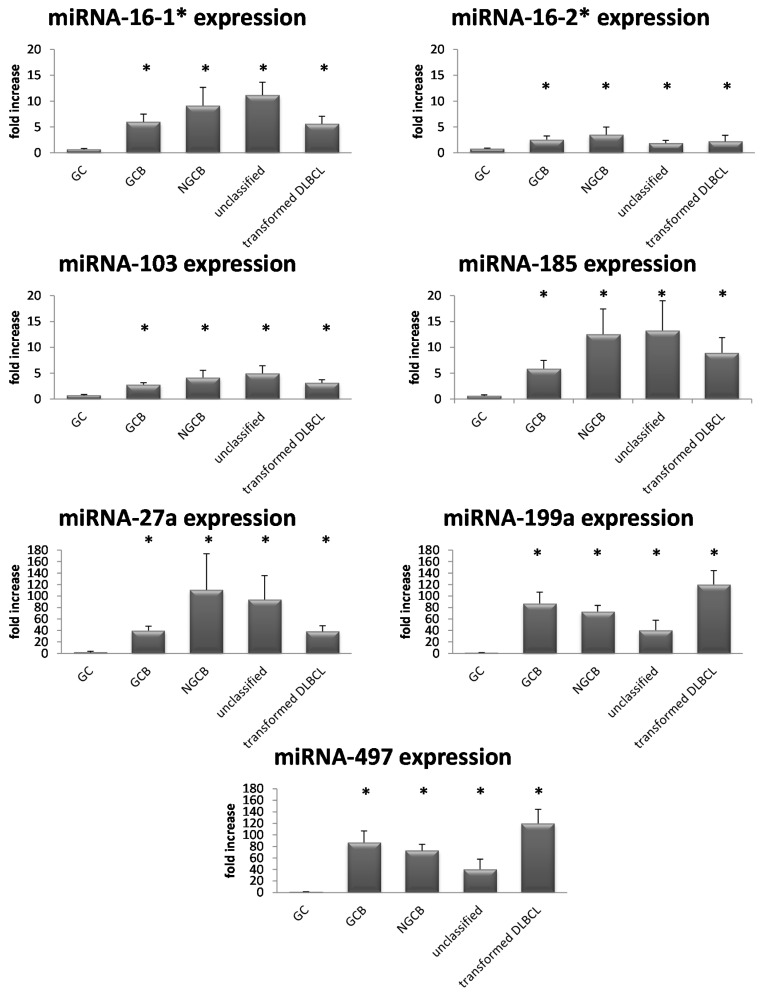
Expression levels of 7 miRNAs (miR-16_1*, miR-16_2*, miR-103, miR-185, miR-27a, miR-199a, and miR-497) according to their subtype. Each bar represents the mean fold increase of miRNA expression ± standard deviation. Statistically significant up-regulation of miRNAs in B-Non Hodgkin Lymphoma (B-NHL) compared to germinal center (GC) cells of at least 6-fold. The comparison of the expression levels was performed by using the Mann–Whitney *U* test. * indicates reduced expression compared with GC cells (*p* < 0.05).

Analysis of expression levels of eleven miRNAs revealed significantly increased expression levels of seven miRNAs (miR-16_1*, miR-16_2*, miR-27a, miR-103, miR-185, miR-199a, and miR-497) in lymphoma specimens compared to germinal center B-cells (Figure 1).

Whereas, for miR-15b_2, miR-16_2, miR-27a*, and miR-98_1 no differential expression was found.

**Figure 2:**
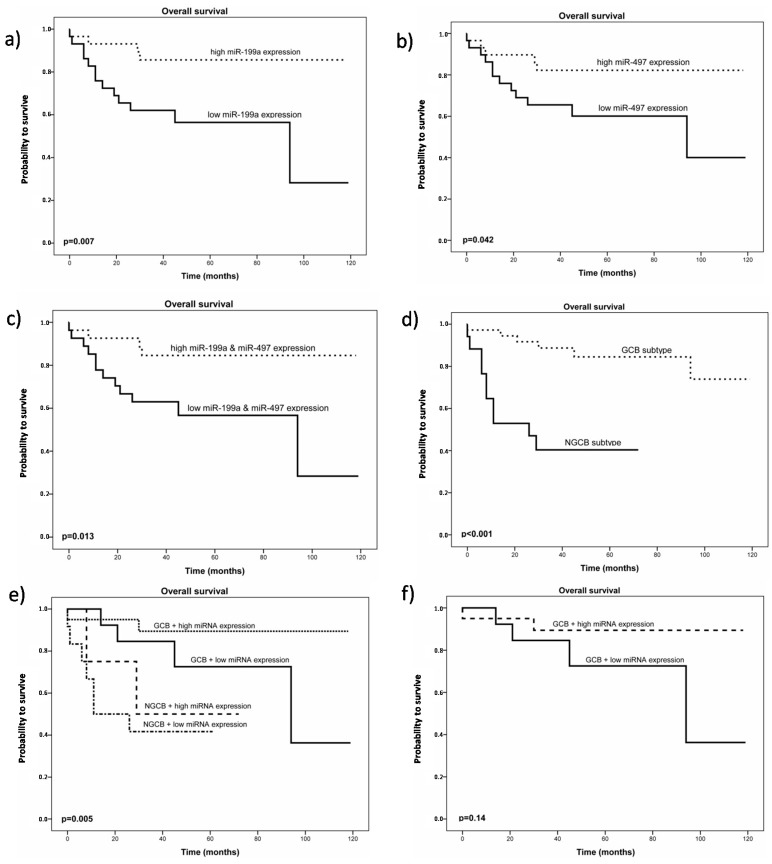
(**a**) Kaplan Meier plot for overall survival in patients with diffuse large B-cell lymphoma (DLBCL) (*n* = 58) stratified by miR-199a expression level. The black dotted line represents patients with high miR-199a expression levels; (**b**) Kaplan Meier plot for overall survival in patients with DLBCL (*n* = 58) stratified by miR-497 expression level. The black dotted line represents patients with high miR-497 expression levels; (**c**) Kaplan Meier plot for overall survival in patients with DLBCL (*n* = 58) stratified by miR-199a and miR-497 expression levels. The black dotted line represents patients with high miR-199a and high miR-497 expression levels; (**d**) Kaplan Meier plot for overall survival in patients with DLBCL (*n* = 55) stratified by their Immunohistochemistry (IHC) profile; (**e**) Kaplan Meier plot for overall survival in patients with DLBCL (*n* = 58) stratified by their IHC profile combined with miR-199a and miR-497 expression levels; (**f**) Kaplan Meier plot for overall survival in patients with DLBCL, germinal center subtype (GCB) (*n* = 38) stratified by miR-199a and miR-497 expression level. The black dotted line represents patients with high miR-199a and miR-497 expression levels.

Analysis of survival data demonstrated that high miR-199a and miR-497 expression levels were associated with better overall survival (OS) in our patient cohort (*p* = 0.007 and *p* = 0.042, Figure 2a,b), whereas no statistical difference regarding overall survival was found for miR-16_1*, miR-16_2*, miR-27a, miR-103, and miR-185. In almost all patients, expression levels of both miRNAs correlated positively to each other (Pearson correlation coefficient 0.678; *p* < 0.001) and improved survival for patients expressing high levels of both miRNAs could also be demonstrated (*p* = 0.013) (Figure 2c).

To evaluate, if these survival effects were not only center specific, we performed analysis of a public available miRNA expression data set of DLBCL patients [22]. Available survival data of 32 patients and their miRNA expression levels confirmed our results of a survival advantage for patients with high expression levels of miR-199a (*p* = 0.01549) (Figure S1). No statistical significant difference in survival was found in this data set for varying miR-497 expression (*p* = 0.64294).

Univariate analysis of clinicopathological parameters for the prediction of overall survival in our patients cohort confirmed the parameters age, R-IPI, cell of origin, stage in addition to the expression values of miRNAs 199a and 497 as prognostic parameters thereby validating our patient collective (Table 2 and Table 3).

**Table 2:** Correlations between miR-199a and miR-497 expressions and clinicopathological parameters.

Clinicopathological Parameters		miR-199a Expression			miR-497 Expression		
		miR-199a Low (*n* = 29)	miR-199a High (*n* = 29)	*p* Value	miR-497 Low (*n* = 29)	miR-497 High (*n* = 29)	*p* Value
Age	≤65	12	20	0.035	12	20	0.035
	>65	17	9		17	9	
Stage	I & II	12	13	0.791	12	13	0.791
	III & IV	17	16		17	16	
R-IPI	1 + 2	14	22	0.043	15	21	0.14
	3 + 4	14	7		13	8	
Subtype	GCB	14	22	0.018	15	21	0.049
	NGCB	13	17		12	5	

R-IPI: revised International Prognostic Index; GCB: germinal center subtype; NGCB: non-germinal center subtype.

**Table 3:** Univariate analysis of clinicopathological parameters for the prediction of overall survival (OS) and disease free survival (DFS) in our patient cohort (*n* = 58).

Parameter	Univariate Analysis OS		Univariate Analysis DFS	
	HR (95% CI)	*p*-Value	HR (95% CI)	*p*-Value
Age at diagnosis (years) <65 ( *n* = 32) ≥65 (*n* = 26)	1 (referent) 4.75 (1.54–14.59)	0.003	1 (referent) 2.53 (1.09–5.87)	0.03
Clinical stage (Ann Arbor) I & II (*n* = 25) III & IV (*n* = 33)	1 (referent) 2.84 (0.93–8.73)	0.049	1 (referent) 3.24 (1.26–8.33)	0.014
R-IPI 1 + 2 (*n* = 36) 3 + 4 (*n* = 21)	1 (referent) 8.6 (2.71–27.28)	<0.001	1 (referent) 3.31 (1.42–7.74)	0.006
Subtype GCB + transformed DLBCL (*n* = 38) NGCB (*n* = 17)	1 (referent) 6.31 (2.13–18.68)	0.001	1 (referent) 2.5 (1.04–5.97)	0.04
miR-199a expression high (*n* = 29) low (*n* = 29)	1 (referent) 0.24 (0.0–0.73)	0.006	1 (referent) 0.47 (0.20–1.1)	0.008
miR-497 expression high (*n* = 29) low (*n* = 29)	1 (referent) 0.36 (0.12–1.01)	0.041	1 (referent) 0.63 (0.27–1.43)	0.265

HR: hazard ratio; CI: confidence intervall; GCB: germinal center subtype; NGCB: non-germinal center subtype; R-IPI: revised International Prognostic Index; OS: overall survival; DFS: disease free survival.

Univariate analysis of the same parameters for disease-free survival showed the same results, except for miR-497 expression. Multivariate analysis was performed regarding all these parameters but lacks explanatory power because of small sample number. The survival advantage for patients with germinal center subtype (GCB) of the Hans algorithm could be confirmed in our cohort (*p* < 0.001) (Figure 2d). In a model, combining the Immunohistochemistry (IHC) profile and miRNA expression levels, patients with GCB subtype and high expression levels of miR-199a and miR-497 had an even better overall survival (Figure 2e). In this small subgroup of GCB subtype (*n* = 38), a trend (*p* = 0.14) for a better OS of patients with high miRNA expression levels could be detected, suggesting a possible tool to further discriminate this subgroup. Together these results indicate that both miRNAs exert biological functions relevant for the prognosis of DLBCL patients after treatment with immunochemotherapy and thus, we proceeded to functionally characterize the properties of miR-199a and miR-497.

### 2.2. Overexpression of miR-199a and miR-497 Causes Higher Chemosensitivity

To clarify a possible biological role of these two miRNAs and to explain the survival differences, we performed a series of* in vitro* experiments by overexpressing miR-199a and miR-497 in four lymphoma cell lines—Namely RI-1, U2932, Karpas-422 and SUDHL4, which expressed both miRNAs at undetectable levels. Transfection of all four cell lines with miRNA mimics resulted in a more than 2000-fold increase of miRNA expression in all cell lines over a period of at least 72 h (data not shown). However, overexpression of the individual miRNAs did not result in any difference in cell viability, cell growth or apoptosis.

**Figure 3:**
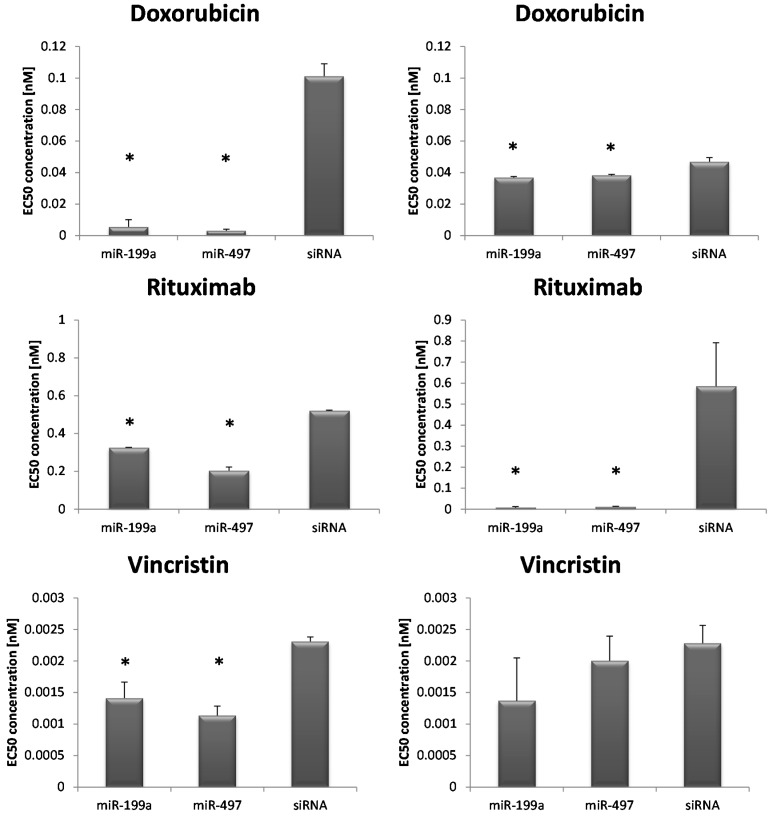
Screening test with 3-(4,5-dimethylthiazol-2-yl)-5-(3-carboxymethoxyphenyl)-2-(4-sulfophenyl)-2H-tetrazolium (MTS) assay. EC_50_ (half maximal effective concentration) concentrations of doxorubicin, rituximab and vincristin of transfected RI-1 (**left column**) and SUDHL4 (**right column**) cells. Each bar represents the mean values of the EC_50_ concentration in nM ± standard deviation of either miR-199a or miR-497 transfected cells or siRNA control, respectively. The comparison of the expression levels was performed by using the Mann–Whitney *U* test. * indicates reduced EC_50_ concentration compared to siRNA (*p* < 0.05).

Additionally, RI-1 lymphoma cells, transfected with either miR-199a or miR-497 and exposed to different concentrations of immunochemotherapy, namely rituximab, vincristin and doxorubicin showed significant lower half maximal effective concentration (EC_50_) concentrations calculated by using 3-(4,5-dimethylthiazol-2-yl)-5-(3-carboxymethoxyphenyl)-2-(4-sulfophenyl)-2H-tetrazolium (MTS) cell growth assays, compared to controls (Figure 3 and Table 4). In a second cell line, SUDHL4, EC_50_ concentrations for doxorubicin and rituximab were also significantly lower in transfected samples, compared to transfection controls (Figure 3 and Table 4). For vincristin, no statistical significant effect could be found in this cell line. No statistical significant difference was found in both cell lines treated with cyclophosphamide (Table 4).

**Figure 4:**
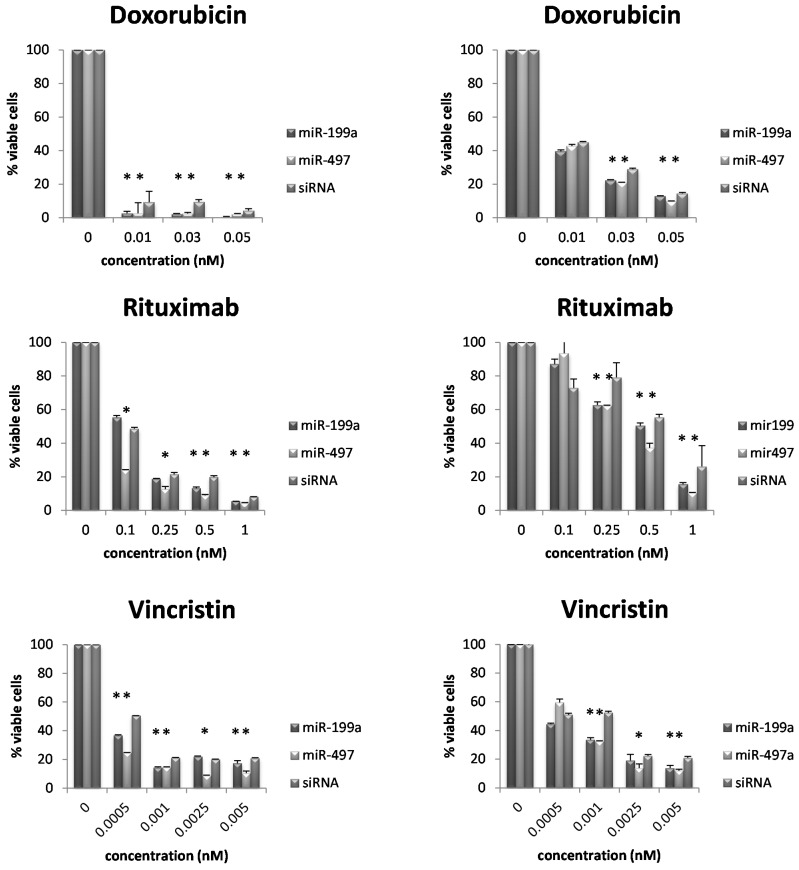
Validation test with Annexin V/7-aminoactinomycin D (7-AAD). Cell viability of RI-1 (**left column**) and SUDHL4 (**right column**) cells treated with doxorubicin, rituximab, and vincristin. Each bar represents the mean cell viability ± standard deviation of either miR-199a or miR-497 transfected cells or siRNA control, respectively. Plotted against the *x*-axis are the used concentrations in nM. Percentage of double negative cells was taken to determine viability. * indicates reduced cell viability compared to siRNA (*p* < 0.05).

To confirm this observation, we determined cell numbers of RI-1 and SUDHL4 cells, either overexpressing miR-199a, miR-497, or siRNA control and treatment with doxorubicin (0.01, 0.03 and 0.05 nM), rituximab (0.1, 0.25, 0.5 and 1 nM) and vincristin (0.0005, 0.001, 0.0025 and 0.005 nM) followed by the determination of Annexin V negative and 7-aminoactinomycin D (7-AAD) negative cell number (viable cells). For these two cell lines, we could confirm a reduced number of viable cells (Annexin V negative and 7-AAD negative) in cells overexpressing either miR-199a or miR-497 compared to negative control siRNA (Figure 4).

**Table 4:** EC_50_ concentrations of the cell lines RI-1 and SUDHL4 after immunochemotherapy treatment (cyclophosphamide, doxorubicin, rituximab, or vincristine) in miRNA overexpressing cells and controls, respectively.

EC_50_ Concentrations (nmol, Standard Deviation)	RI-1				SUDHL4			
	Cyclophosphamid	Doxorubicin	Rituximab	Vincristin	Cyclophosphamid	Doxorubicin	Rituximab	Vincristin
miR-199a	9.65 ± 0.89	0.05 ±0.04	0.33 ± 0.002	0.001 ± 0.000	121.89 ± 10.79	0.03 ± 0.000	0.007 ± 0.005	0.001 ± 0.000
miR-497	30 ± 27.4	0.03 ± 0.01	0.20 ± 0.02	0.001 ± 0.000	9.54 ± 0.98	0.03 ± 0.000	0.011 ± 0.002	0.001 ± 0.002
siRNA	34.85 ± 3.63	1.01 ± 0.08	0.52 ± 0.003	0.002 ± 0.000	2.34 ± 18.5	0.04 ± 0.002	0.585 ± 0.21	0.002 ± 0.002
*t*-test miR-199a* vs.* siRNA	*p* = 0.01	*p* = 0.004	*p* < 0.001	*p* = 0.04	*p* = 0.33	*p* = 0.03	*p* = 0.05	*p* = 0.22
*t*-test miR-497* vs.* siRNA	*p* = 0.83	*p* = 0.003	*p* = 0.002	*p* = 0.009	*p* = 0.4	*p* = 0.04	*p* = 0.05	*p* = 0.50

In summary, these data suggest that overexpression of either miR-199a or miR-497 increases the chemosensitivity of aggressive lymphoma cells towards doxorubicin, rituximab and vincristine representing key components of R-CHOP chemotherapy and might explain the observed survival difference.

## 3. Discussion

miRNAs are crucial players in many pathophysiological processes and with their promising potential as novel diagnostic, as well as predictive markers for therapy, our knowledge regarding their role in the development of solid tumors and in hematologic malignancies is rapidly expanding [23]. The majority of known and characterized miRNAs may function as a tumor suppressor or oncogenes and their respective function depends on the cell type- or tissue specific context [24]. For instance, a tumor suppressive role for miR-16 was identified in breast cancer [25]. On the other hand, miR-27a seems to act oncogenic in gastric cancer by promoting metastasis [26], as well as miR-103, which promotes multidrug resistance [27]. In colorectal-, as well as in prostate, cancer, miR-185 has been identified as a tumor suppressor [28,29,30]. In our study, we identified two (miR-199a and miR-497) of eleven miRNAs as prognostic relevant in DLBCL patients uniformly treated with immunochemotherapy. MiR-199a and miR-497 are mostly found to act tumor suppressive in most solid cancers [31,32,33,34,35,36]. In hepatocellular carcinoma, miR-199a has been found to be down-regulated in nearly all cases investigated, and their tumor suppressive function is exerted partly by their anti-proliferative and anti-growth potential by regulating Hypoxia-inducible factor-1 alpha (HIF-1α) and Serine/threonine-protein kinase PAK 4 (PAK4), as well as by modulating effects via the mammalian target of Rapamycin (mTOR) pathway [31,37,38,39]. Restoring attenuated levels of miR-199a also increased sensitivity to doxorubicin-induced apoptosis [39]. The correlation of miR-199a and the mTOR pathway is also confirmed in human osteosarcoma, in which transfection of precursor miR-199a acts tumor suppressive [33]. In papillary thyroid carcinoma, miR-199a induces lethality via a non-apoptotic form of cell death [32]. On the other hand, NF-κB, one of the key transcription factors in proinflammatory response, could be identified as a crucial target of miR-199a in ovarian cancer cells, in which (IκB kinase β) IKKβ, the direct upstream activator of NF-κB is tightly regulated by this miRNA [40]. Also a tumor-suppressive function of miR-199a, being pro-apoptotic by targeting the MET proto-oncogene and its downstream effector ERK2, was demonstrated in fibroblasts [41]. The same effect of decreased miR-199a expression and therefore pro-oncogenic growth of tumors has also been found in hepatocellular carcinoma [42], renal cancer cells [43], ovarian cancer [44], endometrial cancer [20], and gastric carcinomas [45]. In hematologic malignancies, a regulatory role of miR-199a by negatively affecting cell migration has been identified in multiple myeloma so far [46]. Of possible relevance in lymphomagenesis also the activated mTOR pathway could be identified [20,45]. Furthermore, in comparison of two forms of primary central nervous system (CNS) lymphoma, different expression levels of miR-199a were found [47].

For miR-497, down-regulation was correlated with breast cancer progression, demonstrating a tumor suppressive role [48]. As direct target, the key cell cycle regulator WEE1 has been identified in neuroblastoma, again associated with impaired survival in miR-497 down-regulated samples [49]. Another focus of interest described by Guo* et al.* [36] was the observation of a significant correlation between upregulated insulin-like growth factor 1 receptor and down-regulated miR-497 expression levels in patients with colorectal cancer. In hepatocellular carcinoma, miR-497 appears to act over cell cycle regulation, demonstrating a growth-suppressive activity with induction of Growth 1 (G1) arrest [35].

These reports demonstrate the crucial role of miR-199a and miR-497 in development and progression of cancer. However, since there are currently no systematic studies in aggressive lymphoma patients, we evaluated the role and detailed function of miR-199a and miR-497 and could demonstrate that high expression levels of these two miRNAs are correlated with a better overall survival. Furthermore, we were able to confirm this survival benefit for miR-199a in an independent patient cohort, thus substantiating this novel observation. For miR-497, our results could not be confirmed in an independent cohort, this might be due to the small patient cohort and center-specific differences. To identify the causative pathomechanisms behind this phenomenon, we performed functional analysis in miRNA transfected lymphoma cell lines. Neither of these cell lines showed a different behavior in cell viability or apoptosis by solely overexpression of the respective miRNAs. Nevertheless, after chemotherapy treatment of transfected cells involving the standard immunochemotherapeutical compounds of R-CHOP, we could demonstrate a significant decrease in cell viability in transfected cells, compared to normal controls. First, in a screening test, using MTS assays, we could prove a higher chemosensitivity against doxorubicin, rituximab, and vincristine in cells with upregulated miRNAs, explaining the prolonged survival of these patients. To exclude test-specific biases of the MTS assay, we validated these results in a subsequent experiment, using Annexin V/7-AAD cell viability testing and found again statistical significant reduced cell viability in cells transfected with miRNAs and subsequent chemotherapeutical treatment. The effects of miR-199a/miR-497 overexpression in the two cell lines is quite variable, but may can be explained by their different cell of origin entity and therefore a different chemosensitivity. However, a higher chemosensitivity induced by miRNA up-regulation following therapeutic exposure could be demonstrated and would explain their tumor suppressive role in treated DLBCL patients. This has already been demonstrated by Fornari* et al.* [39] in hepatocellular carcinoma cells (HCC). By restoring attenuated levels of miR-199a in HCC cells they detected an increased sensitivity to doxorubicin-induced apoptosis. In colorectal cancer, Guo *et al.* identified miR-497 overexpression as an inhibitor of cell survival, as well as responsible for increased sensitivity to apoptosis induced by chemotherapeutics [36]. Re-sensitization to chemotherapy by miR-497 overexpression could also be demonstrated in pancreatic cancer, in which low expression levels an adverse prognostic factor [34]. However, such functional characteristic has not been identified in DLBCL yet.

Recently, a number of studies have proposed that circulating miRNAs could be ideal biomarkers for the prediction and prognosis of cancer, furthermore clinical studies have also demonstrated the potential use of miRNAs as promising biomarkers for assessing cancer prognosis [50]. The reasons for disrupted miRNA-mediated regulation of gene expression are not fully understood up to now. In hematological malignancies, various studies discuss the relevance of miRNA target site polymorphisms in the development of cancer, offering the possibility of novel diagnostic and prognostic markers [51]. Dzikiewicz-Krawczyk* et al.* could demonstrate that microRNA-binding site polymorphisms modulate leukemia risk by interfering with the miRNA-mediated regulation [52]. To evaluate these new findings in DLBCL, extended studies are required. Beside miRNAs, the role of long non-coding RNAs have to be elucidated in lymphomagenesis. Their role as tumorsuppressors or oncogenes has been recently reported and there are multiple studies ongoing, investigating their potential as biomarkers and prognosticators [53].

As summary, our findings imply that miR-199a or miR-497 up-regulation significantly increases the probability of patients’ survival by augmenting the chemosensitivity of lymphoma cells. These findings strengthen the understanding of the role of miRNAs in DLBCL development and might eventually lead to a more refined characterization of these miRNAS for future diagnostic and therapeutic application.

## 4. Experimental Section

### 4.1. Samples and Patients

Tumor specimens of 63 patients, including 40 DLBCL samples and 23 transformed DLBCL samples, diagnosed at the Division of Pathology, Medical University of Graz were used. All samples tested were biopsies of various lymph node locations at first diagnosis and only fresh frozen material was used. Using the WHO classification, all samples represent the DLBCL not otherwise specified subtype. IHC profiles according to the Hans algorithms [54] were available for all cases, classifying 15 patients as germinal center subtype (GCB), 17 as non-germinal-center B-cell like, and 8 as non-classifiable according to the Hans algorithms. In these eight cases classification was not possible because of lacking available markers. The 23 transformed DLBCL samples (originated from follicular lymphoma) exhibited the same expression pattern like the GCB-DLCBL therefore they were added to the GCB-DLBCL group for survival analysis [55]. Additionally, 18 samples, including 9 peripheral CD19^+^ B-cells, 5 CD77^+^ germinal center B-cells, and 4 reactive lymphadenopathies served as non-neoplastic controls. For 58 of the 63 patients clinical data were available and characteristics are summarized in Table 1. Patients were treated by standard R-CHOP regimen, post-treatment surveillance included routine clinical and laboratory examination. Follow-up evaluations were performed every three months during the first five years and annually thereafter. Dates of death were obtained from clinical records, the central registry of the Austrian Bureau of Statistics. Clinical data of the patient samples were collected from SAP Medocs. The study was approved by the local ethical committee of the Medical University of Graz (No. 25-434 ex 12/13) in December 2013.

### 4.2. RNA Isolation and Real-Time PCR

Total RNA was extracted using the Trizol method (Invitrogen Life Technologies, Carlsbad, CA, USA). Complementary DNA (cDNA) was synthesized from 500 ng of total RNA using a miScript Reverse Transcription Kit (Qiagen, Venlo, The Netherlands). The resulting 81 samples were analyzed with regard to their expression levels of 11 miRNAs (miR-15b_2, miR-16_1*, miR-16_2, miR-16_2*, miR-27a, miR-27a*, miR-98-1, mir-103a, miR-185, miR-199a, and miR-497) using the miScript system of Qiagen. Expression of the above mentioned miRNAs and 2 additional housekeeping genes (*SNORD68_1* and *RNU6-2_1*) was analyzed in the samples using the LightCycler^®^ 480 (Roche, Basel, Switzerland), with an especially designed protocol. Relative difference of expression was calculated using 2^−ΔΔ*C*t^.

### 4.3. Cell Lines, Culture Conditions, Transfection and Treatments

Moreover, 4 lymphoma cell lines (SUDHL-4, RI-1, U2932, and Karpas) were analyzed with respect to their miRNA expression levels of miR-199a and miR-497. SUDHL-4, RI-1, and U2932 were maintained in Roswell Park Memorial Institute 1640 (RPMI) Medium with 10% fetal bovine serum (FBS), penicillin (50 U/mL), and streptomycin (50 μg/mL). Karpas-422 was maintained in RPMI-1640 with 20% FBS, penicillin (50 U/mL), and streptomycin (50 μg/mL). Cells were periodically checked for mycoplasma by PCR and were found to be negative. These cell lines, obtained from the German Collection of Microorganisms and Cell Cultures (Bonn, Germany), were chosen because of their excellent characterization in literature and their known molecular equality to our entities.

Cells were transfected by electroporation using AMAXA^®^ Cell Line Nucleofector^®^ Kit V (Lonza, Cologne, Germany). The used Nucleofector^®^ programs were G-016 for SUDHL4 and X-001 for Karpas422, RI-1, and U2932. Cells were transfected with either 2000ng siRNA (AllStars Hs Cell Death Control siRNA, Qiagen, Hilden, Germany) or miRNA mimics (Syn-hsa-miR-199a-3p miScript miRNA Mimic, Syn-hsa-miR-497-5p miScript miRNA Mimic; Qiagen). Samples were transferred into 12-well plates containing 1 mL culture medium. Until analysis, cells were incubated in a humidified 37 °C/5% CO_2_ incubator. Success of transfection was measured by Fluorescence activated cell sorting (FACS) analysis, using the LSR II (Becton Dickinson, Franklin Lakes, NJ, USA).

For the treatment experiments, we proceeded with only two cell lines, RI-1 and SUDHL4, in which transfection showed the highest success. After 24 h transfected cells were treated with components of R-CHOP: cyclophosphamide (Baxter Oncology GMBH, Halle, Germany), doxorubicin (Pfizer Service Company BVBA, Zaventem, Belgium), vincristin (Pfizer Italia S.R.L. Nerviano, Italy), and rituximab (Roche Austria GMBH, Vienna, Austria) at concentrations ranging from 0.0005 to 50 nM (Table S1).

### 4.4. Cell Viability and Apoptosis Assay

To determine the number of total and viable cells, counting was performed using the Casy TTC cell counter and analyzer (Roche Diagnostics, Indianapolis, IN, USA).

Cell viability was assessed after 72 h, using the CellTiter 96 Aqueous One Solution Proliferation Assay (Promega, Madison, WI, USA). EC_50_ values were calculated as described [52].

For apoptosis assays, cells were stained with Annexin V-PE/7-AAD with the Annexin V-PE Apoptosis Detection Kit I (Becton Dickinson, Heidelberg, Germany) according to manufacturer’s protocol. Briefly, cells were washed and centrifuged in binding buffer (0.1 M HEPES/NaOH (pH 7.4), 1.4 M NaCl, 25 mM CaCl_2_), and the pellet was resuspended in 5 µL Annexin V-PE, 5 µL 7-AAD (7-amino-actinomycin D) and 200 µL binding buffer, followed by incubation for 15 min at room temperature in the dark. Measurement was performed by flow-cytometer using the LSR II (Becton Dickinson, Heidelberg, Germany). Percentage of double negative cells was taken to determine viability.

### 4.5. Statistical Analysis

The expression of the miRNAs was recorded and analyzed for prognostic relevance. The patients were divided into two groups according to the median expression value and Kaplan–Meier curve analyses were performed together with the log-rank test.

Fisher’s exact test, χ^2^ test, Mann–Whitney test and Student *t* test were used to analyze the association between expression and clinicopathological parameters. Five-year OS was defined as the time (in months) from the date of diagnosis until death due to any cause within the follow-up period. Five-year progression-free survival (PFS) was defined as the time (in months) from the date of diagnosis until the recurrence of the disease being confirmed radiologically or histologically. All these statistical analyses were performed using SPSS V.19.0 software (SPSS, Chicago, IL, USA).

To validate our findings in an external cohort, we performed an analysis of a free available patients’ data set of DLBCL patients, recently published by Caramuta* et al.* [22] along our own data set.

For calculation of EC_50_, SigmaPlot Software (Systat Software, San Jose, CA, USA) was used and EC_50_ values calculated as described [56].

## Supplementary Materials

Supplementary materials can be found at http://www.mdpi.com/1422-0067/16/08/18077/s1.
