# Supplementary material for: miR-199a and miR-497 Are Associated with Better Overall Survival due to Increased Chemosensitivity in Diffuse Large B-Cell Lymphoma Patients
Source: Int J Mol Sci. 2015 Aug 5;16(8):18077–95. doi: 10.3390/ijms160818077 (PMC4581236; doi:10.3390/ijms160818077)
Supplement: Supplementary file 1 [file ijms-16-18077-s001.pdf]

Supplementary Information

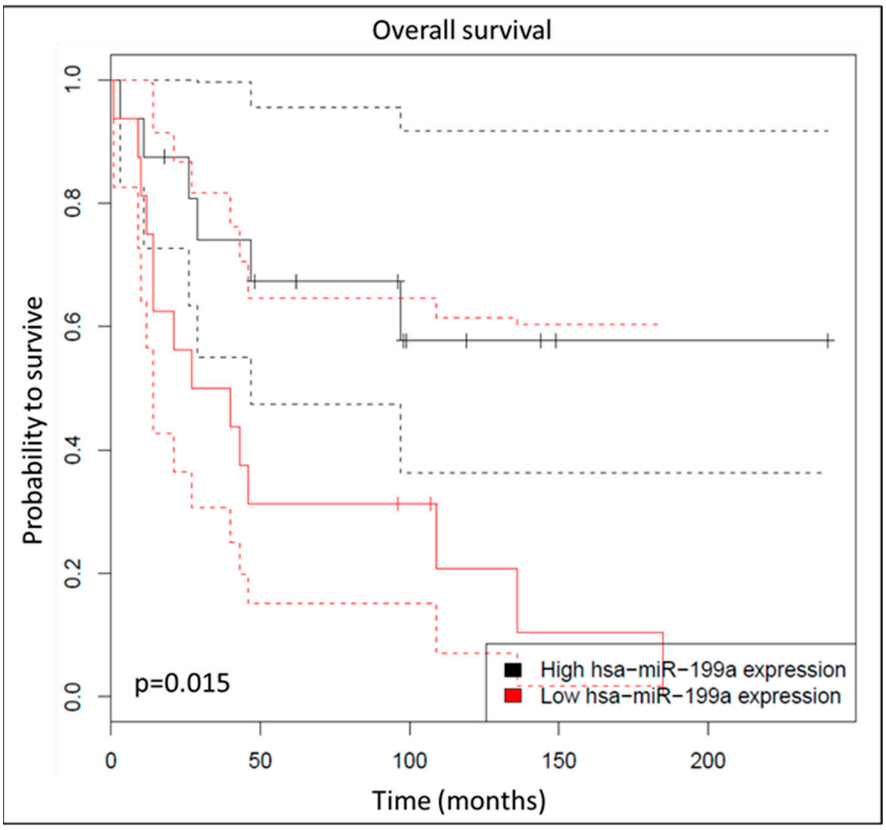

Figure S1. Kaplan Meier plot for overall survival in patients with DLBCL (n = 32) stratified by miR-199a expression level in an analysis of a freely available data set recently published by Caramuta *et al*. [22].

Table S1. Drugs, Doses, and Source used in the study.

Drug	Doses (nM)	Source
Rituximab	0.1, 0.25, 0.5, 1	Roche Austria GMBH, Vienna, Austria
Cyclophosphamide	1, 10, 25, 50	Baxter Oncology GMBH, Halle, Germany
Doxorubicin	0.01, 0.03, 0.05	Pfizer Service Company BVBA, Zaventem, Belgium
Vincristin	0.0005, 0.001, 0.0025, 0.005	Pfizer Italia S.R.L. Nerviano, Italy
